# Open structure ZnO/CdSe core/shell nanoneedle arrays for solar cells

**DOI:** 10.1186/1556-276X-7-516

**Published:** 2012-09-20

**Authors:** Yanxue Chen, Lin Wei, Guanghua Zhang, Jun Jiao

**Affiliations:** 1 School of Physics and State Key Laboratory of Crystal Materials, Shandong University, Jinan, 250100, People's Republic of China; 2 School of Information Science and Engineering, Shandong University, Jinan, 250100, People's Republic of China; 3 Physics Department, Portland State University, PO Box 751, Portland, OR 97207, USA

**Keywords:** ZnO, CdSe, nanoneedles, solar cells

## Abstract

Open structure ZnO/CdSe core/shell nanoneedle arrays were prepared on a conducting glass (SnO_2_:F) substrate by solution deposition and electrochemical techniques. A uniform CdSe shell layer with a grain size of approximately several tens of nanometers was formed on the surface of ZnO nanoneedle cores after annealing at 400°C for 1.5 h. Fabricated solar cells based on these nanostructures exhibited a high short-circuit current density of about 10.5 mA/cm^2^ and an overall power conversion efficiency of 1.07% with solar illumination of 100 mW/cm^2^. Incident photo-to-current conversion efficiencies higher than 75% were also obtained.

## Background

Since the first report on the dye-sensitized solar cell by O'Regan and Grätzel in 1991 [1], a great number of photovoltaic devices based on nanostructures have been proposed or developed, such as nanostructured dye-sensitized cells [2,3], extremely thin absorber (ETA) cells [4], quantum dot cells [5], nanowire array cells [6], organic/inorganic nanostructured cells [7], and III-VI quantum ring solar cells [8]. Nanostructured solar cells have several advantages over conventional bulk and thin film solar cells: large surface area, high efficiency for light harvesting, less expensive materials, and low process cost.

The two most frequently used window materials in nanostructured solar cells are highly porous nanocrystalline TiO_2_ and highly textured ZnO nanorod arrays. Porous nanocrystalline TiO_2_ particles can provide a large surface area for the absorber material. However, their slow trap-limited diffusion process and short effective diffusion length of electrons are big obstacles in making more efficient cells. ZnO nanowires have higher carrier concentration and electron mobility which favor the electron transport to the collection electrode. As the nanowires are not in direct contact with each other, the electrons transport only along the nanowire axis without any lateral transport, which will reduce the non-radiative recombination and carrier scattering loss dramatically. Solar cells sensitized by organic dye absorbers have shown impressive results, although their long-term stability and bandgap controllability need to be improved further. On the other hand, inorganic narrow bandgap semiconductors, such as Ag_2_S [9], In_2_S_3_[10], CdS [11], CuInS_2_[12], and CdSe [13], are also promising candidates as sensitizers for nanostructured solar cells.

It has been postulated that ZnO/CdSe can form a type II heterojunction which will accelerate the separation of photoexcited electron–hole pairs and improve the efficiency of solar cells. In a previous study, Leschkies et al. fabricated CdSe quantum dot sensitized ZnO nanowire solar cells [14]. They recorded a power conversion efficiency of 0.4% and a short-circuit current density of 2.1 mA/cm^2^, which are still low compared with those of dye-sensitized solar cells. Lévy-Clément et al. prepared a nanostructured ZnO/CdSe/CuSCN ETA solar cell [15,16], and a high energy conversion efficiency greater than 2% was demonstrated under a 340-W/m^2^ illumination using a halogen lamp. However, they did not report the energy conversion efficiency under the air mass (AM)1.5 full sun intensity. Luan et al. reported a CdS/CdSe co-sensitized solar cell using a facile solution growth which resulted in a power conversion efficiency of approximately 1% with a fill factor of 0.55 [17]. Until now, there have been only a few reports published concerning ZnO/CdSe nanostructure-based solar cells. The mechanisms of such structures have not been systemically studied, and more fundamental researches should be conducted to provide further understanding of the electronic transporting process in these nanostructures. Herein, we reported the fabrication and characterization of open structure ZnO/CdSe core/shell nanoneedle array-based solar cells. High short-circuit current densities and power conversion efficiencies were obtained, which provided significant insight as to how to improve the photovoltaic performance of this type of solar cell.

## Methods

### Growth of ZnO nanoneedle arrays by solution deposition

ZnO nanoneedle arrays were grown using solution deposition method [18] on fluorine-doped SnO_2_ (SnO_2_:F) substrate covered with a ZnO seed layer. The ZnO seed layer was formed by spin coating a solution of zinc acetate and ethanolamine in 2-methoxy-ethanol at 3,000 rpm, followed by annealing in a furnace at 400°C for 1 h. Seeded substrates were placed vertically in aqueous solutions containing 20 mM zinc nitrate, 20 mM hexamethylene-tetramine, and 125 mM 1,3-diaminopropane at 70°C for 12 h. The sample containing ZnO nanoneedle arrays was rinsed with deionized water thoroughly and annealed at 500°C for 1 h to remove any residual organics and to improve the crystalline structure.

### Deposition of CdSe shell layer using electrochemical technique

A CdSe coating layer was electrochemically deposited at room temperature on the ZnO nanoneedle arrays from an aqueous selenosulfate solution [19]. A two-electrode electrochemical cell was used with the ZnO nanoneedle arrays as the cathode and a Pt wire as the counter electrode. CdSe was deposited under galvanostatic conditions with a current density of 1 mA/cm^2^ and a charge density of 0.25 C/cm^2^. The samples were annealed at 400°C for 1.5 h to increase the mean grain size, which can help to reduce the negative effects of grain boundary trap states.

### Characterization of ZnO nanoneedle arrays and ZnO/CdSe core/shell nanostructures

The crystal structure of the samples was examined by X-ray diffraction (XD-3, PG Instruments Ltd., Beijing, China) with Cu-K*α* radiation (*λ* = 0.154 nm) at a scan rate of 2° per min. X-ray tube voltage and current were set at 40 kV and 35 mA, respectively. The morphologies of the different nanostructures were investigated by scanning electron microscopy (SEM) (FEI Sirion, FEI Company, Hillsboro, OR, USA). The high-resolution transmission electron microscopy (HRTEM) images were taken with a Technai F-20 microscope (FEI Company, Hillsboro, OR, USA) at an acceleration voltage of 200 kV. The HRTEM specimens were prepared by drop casting the sample dispersion onto copper grid with holey carbon film and were dried under room temperature. The room temperature photoluminescence (PL) spectra of the ZnO/CdSe core/shell nanostructures were measured by exciting the samples with a YAG solid state laser at a wavelength of 532 nm. The UV-visible absorption spectra were obtained using a UV-visible spectrometer (TU-1900, PG Instruments, Ltd., Beijing, China).

### ZnO/CdSe core/shell solar cell assemble and performance measurement

The solar cells were assembled using the ZnO/CdSe core/shell nanoneedle array-covered SnO_2_:F glass as the photoanode and a SnO_2_:F glass coated with a thin platinum layer (approximately 10 nm) as the counter electrode. A 100-μm-thick spacer was sandwiched between these two electrodes to prevent electrical shorts. A polysulfide electrolyte containing 1 M Na_2_S and 1 M S was injected into the space between the nanoneedle arrays and the platinized SnO_2_:F cathode to complete the cell assembly. The solar cell current–voltage characteristics were measured using a Keithley 2400 sourcemeter (Keithley Instruments Inc., Cleveland, OH, USA) while illuminating the solar cells with a solar simulator (model 94022A, Newport, OH, USA) at one sun (AM1.5, 100 mW/cm^2^). The measurements were carried out with respect to a calibrated OSI standard silicon solar photodiode. The incident photon-to-current conversion efficiency (IPCE) measurements were carried out with a custom measurement system consisting of a 150-W Xe lamp (LSH-X150, Zolix, Beijing, China), a monochromator (7ISW30, 7 Star Optical Instruments Co., Beijing, China) and a sourcemeter (2400, Keithley Instruments Inc.).

## Results and discussion

### Morphology and crystal structure of ZnO nanoneedle arrays and ZnO/CdSe core/shell nanostructures

**Figure 1:**
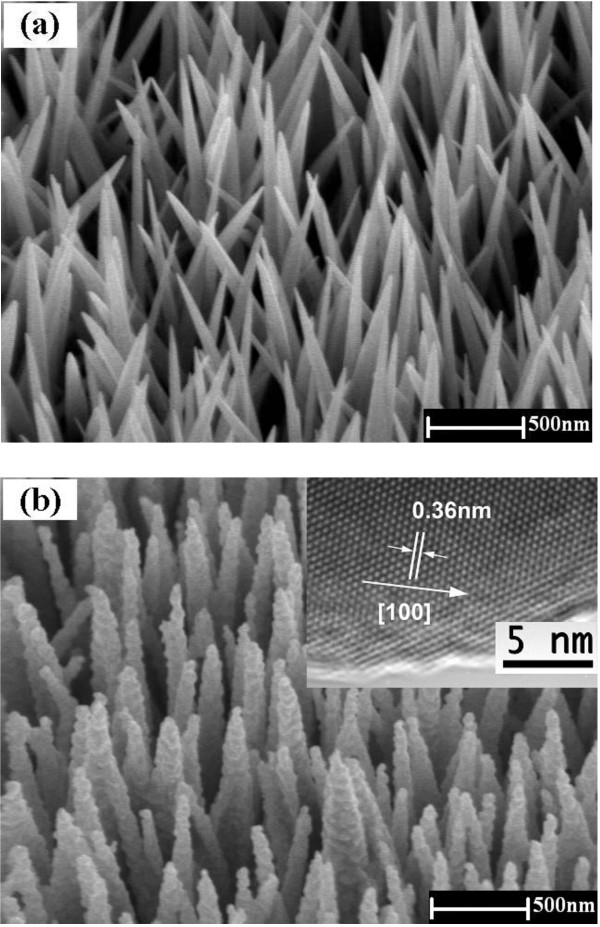
** Typical SEM images of ZnO nanoneedle arrays and ZnO/CdSe core/shell nanostructures.** (**a**) SEM image (40° tilted) of a ZnO nanoneedle array grown on SnO_2_:F substrate by solution method. The average length of the nanoneedle is about 4 to 5 μm. The diameter to the tip is 10 nm, and the diameter to the base is 200 nm. (**b**) SEM image (40° tilted) of a ZnO/CdSe core/shell nanoneedle array coated by electrodeposition. Inset: HRTEM image of a CdSe grain.

Figure 1a shows an image of an as-grown ZnO nanoneedle array taken by a field emission scanning electron microscope. The SEM image clearly shows that ZnO nanoneedles with sharp tips are grown vertically on the SnO_2_:F substrate. Further analysis indicates that the average length of the nanoneedles is about 4 to 5 μm, and the diameters are 10 nm at the tip and 200 nm at the base. This nanoneedle array presents an easily accessed open structure for CdSe deposition and higher hole transferring speed for the whole solar cell. No significant changes in nanoneedle array morphology were observed after annealing at 500°C. After the deposition of CdSe layer and annealing, a conformal and uniform coverage of all nanoneedles can be seen in Figure 1b. The oval grains of CdSe form with a diameter of about several tens of nanometers are distributed uniformly over the entire nanoneedle (with no shadowing effects at the base which would otherwise be more prevalent with PVD methods). The single crystallinity of CdSe grains was confirmed by HRTEM study, as displayed in the inset of Figure 1b. The lattice spacing obtained from this HRTEM image was 0.36 nm, which corresponds to the separation between the {100} lattice planes of wurtzite CdSe.

**Figure 2:**
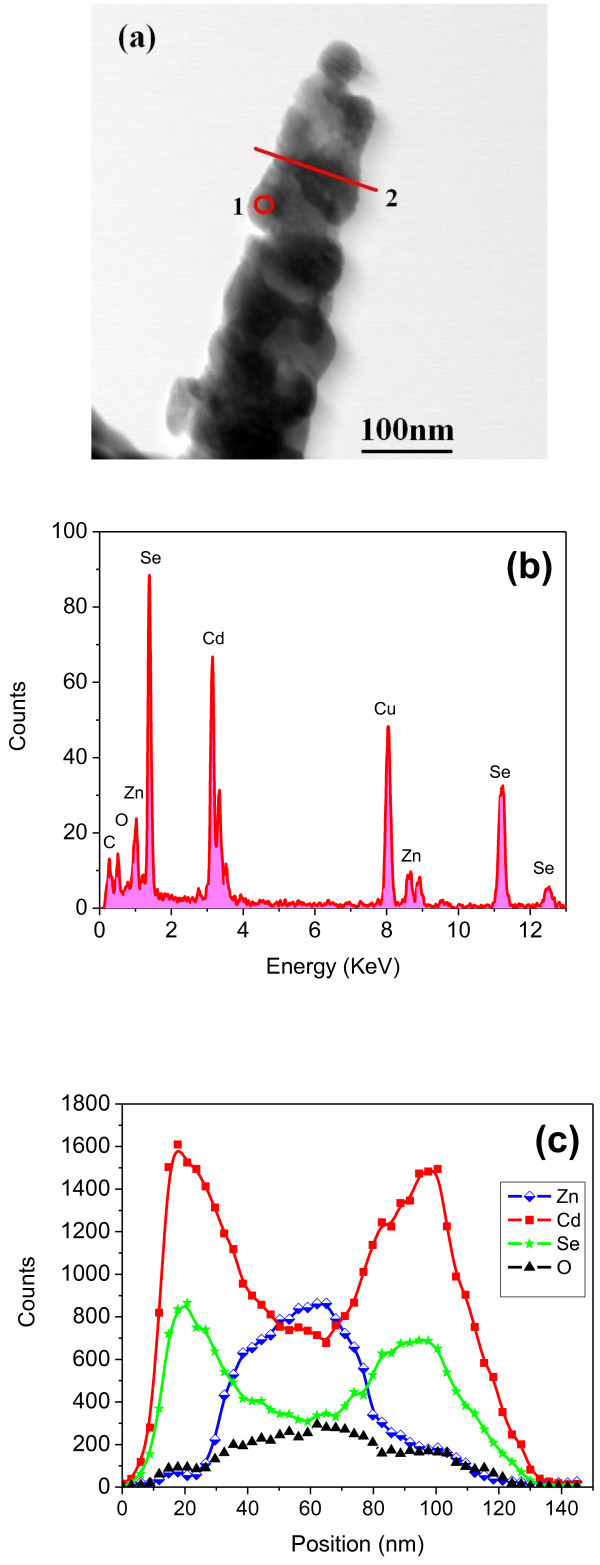
** Morphology and atomic constituent spatial distribution of the ZnO/CdSe core/shell nanostructures.** (**a**) Bright field STEM image of the ZnO/CdSe core/shell nanoneedle. (**b**) EDX spectra of the CdSe shell layer as indicated by point 1 in (a). (**c**) EDX nanoprobe line scan of the elements Zn, Cd, and Se across the ZnO/CdSe core/shell nanoneedle as indicated by line 2 in (a).

The morphology and spatial distributions of the atomic constituents of the ZnO/CdSe core/shell nanoneedles were further investigated using a Technai F-20 TEM equipped with an energy dispersive X-ray (EDX) spectrometer and operated in a scanning transmission electron microscopy (STEM) mode. A low-magnification STEM image of a core/shell nanoneedle is given in Figure 2a, which shows that the CdSe grain size ranges in between 50 to approximately 90 nm. Typical EDX results for the shell layer (corresponding to point 1 in Figure 2a) are displayed in Figure 2b, confirming that the shell layer consists of mainly Cd and Se elements. The weak Zn and O peaks in the point spectrum can be attributed to the interaction volume of the electrons, and the C and Cu peaks are from the TEM grid. An EDX line scan along line 2 in Figure 2a was conducted to demonstrate the CdSe coverage on the ZnO nanoneedle. As shown in Figure 2c, the ZnO nanoneedle is homogeneously coated with CdSe shell layer.

### Optical properties of the ZnO/CdSe core/shell nanostructures

**Figure 3:**
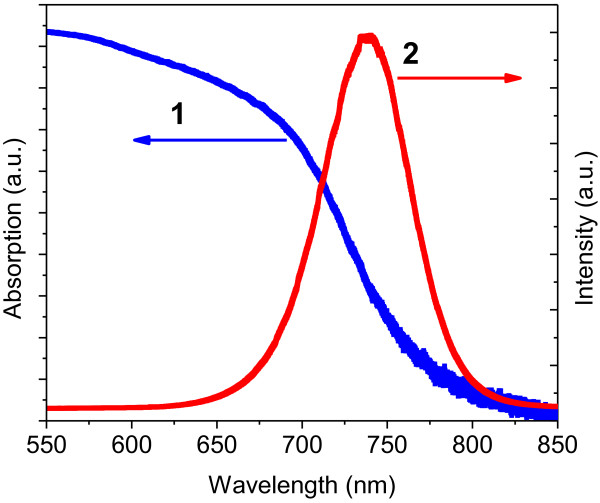
Optical absorption (curve 1) and photoluminescence (curve 2) spectra of ZnO/CdSe core/shell nanoneedle arrays.

The optical properties of the ZnO/CdSe core/shell nanostructures were investigated by absorption and PL measurements. Figure 3 shows the absorption and PL spectra of the ZnO/CdSe core/shell nanoneedle arrays. An optical bandgap of 1.71 eV is estimated for the CdSe layer from the absorption spectra, which is in good agreement with that of bulk CdSe. As the size of the CdSe grains is well above the CdSe Bohr exciton diameter (approximately 3 nm), no obvious blueshift caused by quantum confinement is observed. Similar to the cases of ZnO/ZnSe core/shell nanowires, a significant optical absorption is observed at wavelengths longer than the CdSe bandgap, which may arise from a spatially indirect transition or an interfacial transition coupling a hole state in CdSe shell with an electron state in the ZnO core. Strong bandgap excitonic emission at 1.68 eV upon excitation with a 532-nm laser is observed at room temperature. This high PL intensity indicates the high interior crystal quality and low defects of the CdSe shell layer, which is essential to reduce the recombination of the excited electron–hole pairs and increase the photocurrent of the solar cells. The high interior crystal quality of the CdSe shell layer is also confirmed by its HRTEM image (inset of Figure 1b).

### Photovoltaic performance of ZnO/CdSe core/shell solar cells

**Figure 4:**
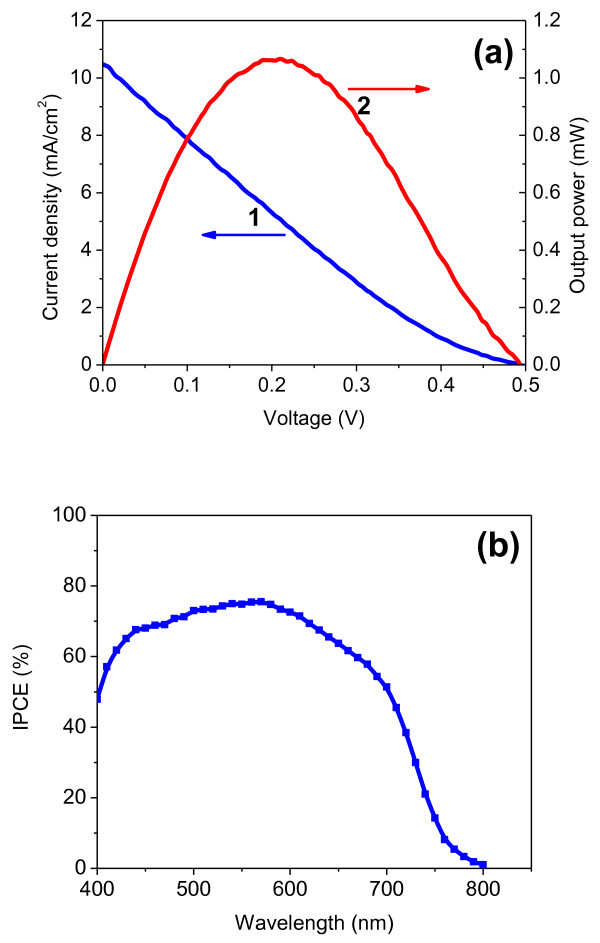
** Photovoltaic performance of ZnO/CdSe core/shell solar cells.** (**a**) Photocurrent density and voltage characteristic (curve 1) and the power output (curve 2) of the ZnO/CdSe core/shell nanoneedle array-based solar cells under 100 mW/cm^2^ of simulated AM1.5 spectrum. (**b**) IPCE spectra of the same solar cell.

Current and voltage characteristics of the ZnO/CdSe core/shell nanoneedle array-based solar cell were measured under 100 mW/cm^2^ of simulated sunlight illumination (AM1.5). As shown in Figure 4a, an open voltage of 0.5 V, a short-circuit current density of 10.5 mA/cm^2^ and an overall energy-conversion efficiency of 1.07% were generated. These values are an improvement over recently reported CdSe quantum dot sensitized ZnO nanowire solar cells [14]. These promising improvements can be attributed to three important factors of the ZnO/CdSe core/shell nanoneedle-based solar cell: strong light absorption in a wider wavelength range; higher CdSe coverage on ZnO surface, and direct contact between CdSe and ZnO without any interlinking material. Figure 4b shows the IPCE spectrum of the same solar cell used to measure the *IV* characteristics. From the spectrum, a high IPCE value above 50% is measured in the wavelength range of 400 to approximately 700 nm with the highest value of 76% at 570 nm. This wavelength range is in good correlation with the energy range of the sunlight spectrum at the Earth's surface where the flux is maximal. The IPCE values decrease steeply at wavelength above 700 nm, which are matched well with those of the corresponding transmission spectrum in Figure 3 (curve 2).

From the high short-circuit current density and the IPCE values, we can conclude that the ZnO/CdSe interface forms an ideal type II heterojunction with suitable band alignment, which is essential to efficient charge transfer. ZnO nanoneedles have good electron conductivity and form very open structures, which is advantageous over the short effective diffusion length of electrons and the diffusion problems associated with the redox couples in the porous TiO_2_ network. The short-circuit current density can be further improved by increasing the length of the ZnO/CdSe core/shell nanoneedles. The drawback limiting the energy conversion efficiency of this type of solar cells is a rather poor fill factor of 0.22, which limits the energy conversion efficiency. This low fill factor may be ascribed to the lower hole recovery rate of the polysulfide electrolyte, which leads to a higher probability for charge recombination [20]. Although the I^−^/I_3_^−^ redox couple has ideal kinetic properties in regeneration of the oxidized dye and in inhibition of the recombination of an excited electron with the electrolyte, it is corrosive to the CdSe semiconductor, which will cause a rapid degradation of the solar cell performance. To further improve the efficiency of these nanoneedle array solar cells, a new hole transport medium with suitable redox potential and low electron recombination at the semiconductor and electrolyte interface should be developed. Recently Li et al. reported a very high fill factor of 0.89 in CdS quantum dot sensitized solar cells based on a modified polysulfide electrolyte [21]. If this electrolyte is suitable for our ZnO/CdSe core/shell solar cells, a much better photovoltaic performance can be expected. Moreover, as reported by Soel et al., other contributions such as the counter electrode material may also have an influence in the fill factor [22].

## Conclusions

In summary, we have prepared open structure ZnO/CdSe core/shell nanoneedle arrays on SnO_2_:F glass by solution deposition and electrochemical techniques. Optical measurements indicate that these nanostructures are very favorable for the use in photovoltaic devices. Nanoneedle array-based solar cells were assembled using a polysulfide electrolyte. A much higher short circuit current and IPCE (76%) are obtained in these solar cells, showing a promising alternative to existing dye-sensitized solar cells.

## Competing interests

The authors declare that they have no competing interests.

## Authors' contributions

YC carried out the preparation of ZnO/CdSe core/shell samples and solar cell devices and drafted the manuscript. LW conducted the absorption and the photoluminescence spectra measurements. GZ participated in the current density and voltage performance measurement and analysis. JJ carried out TEM and HRTEM characterizations and revised the manuscript. All authors read and approved the final manuscript.
